# Survival Analysis and Risk Factors of Pulpectomy among Children with Severe Early Childhood Caries Treated under General Anesthesia: A Retrospective Study

**DOI:** 10.3390/ijerph20021191

**Published:** 2023-01-09

**Authors:** Shu-yang He, Jin-yi Li, Shan-shan Dai, Yu-hui Yang, Yi-feng Wen, Qing-yu Guo, Fei Liu

**Affiliations:** 1 Key Laboratory of Shaanxi Province for Craniofacial Precision Medical Research, College of Stomatology, Xi’an Jiaotong University, Xi’an 710004, China; 2 Faculty of Dentistry, The University of Hong Kong, Hong Kong, China; 3 Department of Pediatric Dentistry, Affiliated Stomatology Hospital of Xi’an Jiaotong University, Xi Wu Road No. 98, Xi’an 710041, China; 4 Department of Epidemiology and Biostatistics, School of Public Health, Xi’an Jiaotong University Health Science Center, Xi’an 710061, China; 5 Department of Endodontics, Affiliated Stomatology Hospital of Xi’an Jiaotong University, Xi’an 710004, China

**Keywords:** retrospective, pulpectomy, survival analysis, primary tooth

## Abstract

Objectives: This study aims to retrospectively evaluate the survival rate of pulpectomy performed under dental general anesthesia (DGA) through long-term follow-up and to explore the risk factors associated with treatment failure. Methods: The medical records of the children who were diagnosed with S-ECC and received pulpectomy treatment under general anesthesia (GA) from 1 August 2014 to 1 December 2019, in the Stomatological Hospital of Xi’an Jiaotong University, were collected. Two dentistry postgraduates extracted the necessary information and filled in a predesigned excel form. Survival analysis was performed using the Kaplan-Meier method. The shared frailty model was used to explore possible factors affecting the success rate of pulpectomy in primary teeth. Results: A total of 381 children (mean age 3.49 ± 0.90) with S-ECC and 1220 teeth were included in the study, including 590 primary anterior teeth and 630 primary molars. The overall 35-month survival rate was 38.5%, which was 52.9% for anterior teeth and 31.1% for molars. The overall median survival time was 31 months, in which anterior teeth were 35 months and molars were 26 months. The older the children were, the greater the risk of treatment failure (HR 1.56, 95% CI 1.09, 2.24). The risk of pulpectomy failure of primary molars was 1.9 times that of primary anterior teeth (95% CI 1.36, 2.65) and the teeth with abnormal radiological findings before treatment was 1.41 times higher than that of teeth without imaging abnormalities (95% CI 1.74, 3.36). Conclusion: The survival rate of primary tooth pulpectomy is acceptable but decreased gradually with time. The failure rate of pulpectomy in primary molars is higher than that of primary anterior teeth. When the primary caries has extended to the pulp and resulted in a nonvital lesion, pulpectomy could be an option for maximum retention of the primary tooth.

## 1. Introduction

Caries in primary teeth are one of the most common health conditions among children globally and locally across a majority of world regions [1]. Among the different types of caries in primary teeth, early childhood caries (ECC) and severe early childhood caries (S-ECC) have more profound societal impacts and pose a substantial public health concern [2]. The global prevalence of ECC ranges from 16% to 89% [3], in which S-ECC accounts for a large proportion [4,5]. Moreover, a high portion of the primary tooth caries were untreated dental caries [4,5]. Primary dental caries develop rapidly but can be easily neglected by parents, resulting in delayed or missed treatment. In the long term, untreated tooth decay may impact children’s systemic health, leading to conditions such as development deficits and psychological problems [6]. The local effect of the untreated carious lesions include pulpal involvement, periapical lesions, abscess, and sinus tract [7]. These constitute substantial direct and indirect costs to children, families, and society, and significantly impacts children’s quality of life.

Regarding lesions involving the pulp, partial pulpotomy, pulpectomy, and tooth extraction are all available options. The American Academy of Pediatric Dentistry (AAPD) states that when there is enough evidence, children should be subjected to nonvital pulp treatment [8]. It is the only treatment to retain the teeth with irreversible pulpitis and periapical periodontitis. With the widespread application and ongoing research on primary tooth pulpectomy, some studies have shown that the treatment has a better prognosis, but definitive conclusions remain elusive. A negative attitude toward pulpectomy in primary teeth is mostly due to the potential risk of damage to the successor permanent tooth buds and difficulty in cleaning and shaping root canals [9].

Meanwhile, there are various potential factors associated with the failure that cannot come into consistency [8,10,11]. The success rate may vary depending on the etiology of lesions, preoperative pulp status, follow-up duration, filling materials, restorations, and clinicians. However, there is currently a lack of comprehensive and longitudinal evaluations of factors influencing the treatment outcome of pulpectomy in primary teeth.

Compared with other endodontic treatments in pediatric dentistry, pulpectomy is more complicated and requires a higher degree of cooperation. When performed under dental general anesthesia (DGA), pulpectomy of the primary tooth is a safe and effective procedure that avoids the interference of children’s uncooperative behaviors [12]. Many studies have shown significant satisfaction and high acceptance of the treatment outcome of DGA [13,14].

Therefore, this study aims to retrospectively evaluate the survival rate of pulpectomy performed under DGA through long-term follow-up and to explore the risk factors associated with treatment failure while controlling for potential confounders such as children’s level of cooperation and contamination during the follow-up visit.

## 2. Materials and Methods

This study was approved by the Medical Ethics Committee of Xi’an Jiaotong University (xjkqll (2021) NO. 10).

### 2.1. Participants

The participants were children diagnosed with S-ECC without systemic illness who underwent DGA in the Department of Pediatric Dentistry, Stomatology Hospital of Xi’an Jiaotong University from 1 August 2014 to 31 December 2019. The participants’ medical records should be complete, and their follow-up period should last at least six months. Radiographs of the tooth under treatment before the operation or within two weeks after the treatment, together with at least one radiograph in the follow-up period, were needed.

The inclusion and exclusion criteria of the tooth are as follows.

Inclusion criteria:

(1) Diagnosed with irreversible pulpitis, pulp necrosis, or periapical periodontitis but reserved;
(2) Without internal resorption or radiolucency surrounding the tooth apex. For teeth with physiological or pathological root resorption, resorption should be limited to less than 1/3 of the root length.

Exclusion criteria:

(1) The crown is seriously damaged and cannot be restored;
(2) Root resorption involving more than 1/3 of the root length;
(3) Inflammation has affected the permanent teeth;
(4) Traumatic injury to the treated tooth during the observation period.

### 2.2. Pulpectomy Procedure

All operations were performed by dentists with at least an associate professorship and more than 15 years of experience and they were familiar with the practice process. All of the teeth were isolated by the rubber dam under the DGA. After removing all of the carious lesions, the pulp chamber was accessed by the high-speed handpiece. The pathological pulp was cleaned with barbed broaches. Root canal preparation was performed using manual (Mani #15 or #20 K files, MANI Inc. Tochigi, Japan) and a rotary (Protaper Universal, Dentsply Maillefer, Ballaigues, Switzerland) instrument. The canal enlargement was performed with ProTaper Universal instruments F1 and F2. The working length was determined by apex locators (ProPex Pixi, Dentsply Maillefer, Ballaigues, Switzerland) and practitioners’ experiences. The root canal was alternatively irrigated with 1.25% sodium hypochlorite and 2% chlorhexidine, and normal saline was used between the use of those two solutions. Sterilized paper point was used to dry the root canals, and the canals were obturated by the root canal filling paste. There are two kinds of filling pastes in clinical practice, zinc oxide eugenol paste (ZOE, Associated Dental Products Ltd., Purton, UK) and Vitapex (Neo Dental Chemical Products Co. Ltd., Tokyo, Japan). The decision on which type to use was made by the practitioners. The filling materials were sent into the root canal by lentuo spirals until there was obvious overflow at the root canal orifice. The choice of the restoration method depended on the size of the defective area and tooth position. The preformed metal crown (PMC, stainless steel crown, SSC) with GIC was routinely selected for molars. For anterior teeth, a strip crown with resin composite (Filtek Supreme Flowable Restorative, 3M ESPE, St. Paul, MN, USA) or direct resin composite (Filtek Z350 XT Universal Restorative, 3M ESPE, St. Paul, MN, USA) was used. The 2-step etch-and-rinse adhesive (Single bond, 3M ESPE) was adopted during the restoration.

### 2.3. DGA Treatment Requirement

All treatment procedures were followed and adjusted according to related guidelines and textbooks. All of the children who underwent DGA treatment were required to follow-up two weeks later the DGA treatment and subsequently every three to six months. No X-ray or clinical examination was conducted immediately after the operation. The patient was required to take a radiograph at the first follow-up visit. The other radiographs were taken every three to six months for teeth that underwent pulpectomy therapy. The radiographs were taken by the experienced technician and adopted by bisected angle technique.

Additionally, parents are advised to prepare non-prescription NSAIDS drugs. It can be taken for one to three days if fever occurs after DGA treatment. Oral health education was conducted for guardians and children at every follow-up visit, and fluoride application was carried out regularly every 3–6 months.

### 2.4. Criteria for the Outcome during the Follow-Up Examinations

The outcome was evaluated both clinically and radiographically.

Clinical criteria for success:

(1) No spontaneous pain or sensitivity to percussion;
(2) The surrounding soft tissues are healthy, or the sinus tract and abscess recovered;
(3) No abnormal mobility;
(4) Has physical function.

Radiographic criteria for success:

(1) The range of low-density shadows in the root area becomes small or disappears;
(2) The bone around the permanent tooth is intact;
(3) No pathological lesion presented in the successor permanent teeth;
(4) No pathological root resorption is observed.

The treatment is considered an overall failure if any one of the above items (regardless of clinical or radiological items) is not met or the primary teeth are lost prematurely.

### 2.5. Data Extraction

Data regarding (1) general information: case number, sex, and age of the patients; (2) preoperative information of teeth: tooth type, arch location, surfaces affected by caries, mobility, gingival condition, and radiological evaluation; (3) treatment information: root canal obturation materials, degree of root canal filling, and the restoration method; and (4) follow-up information: date, symptoms and signs, clinical examination, and radiographic evaluation were extracted independently by two paediatric dentistry postgraduates using a predesigned form in Microsoft Excel. 

### 2.6. Consistency Test

Clinical evaluation is based on the medical record. The radiographic evaluation was independently evaluated by the two postgraduates. A professor resolved any disagreements between the two postgraduates. A pilot test was conducted on 30 images to estimate the consistency. The internal consistency (over two weeks) was 0.92 and 0.96, respectively, and the external consistency between them was 0.90.

### 2.7. Statistical Analysis

SPSS software (22.0 IBM SPSS statistics, Armonk, NY, USA) was used to calculate the mean and standard deviation (SD) and the frequency. R statistical computing software (version 4.04, Vienna, Austria) was used to estimate the survival rates at different times after treatment by the Kaplan-Meier method (K-M method). The annual mean failure rate was calculated according to the following formula [15], where *y* is the annual average failure rate and *x* is the failure rate in the *z*th year.

$${\left(1-y\right)}^{z}=1-x$$

The shared frailty model is a proportional hazards model with random effects. The model was constructed for survival analysis and used to explore factors affecting the efficacy of pulpectomy in primary teeth. The significance level was taken as 0.05.

## 3. Results

From 1 August 2014 to 31 December 2019, a total of 1931 children underwent DGA in the Department of Pediatric Dentistry of Stomatology Hospital of Xi’an Jiaotong University, and 1671 (86.54%) received pulpectomy treatment. A total of 6185 teeth were treated with pulpectomy, with an average of 3.70 ± 2.53 per child. According to the inclusion and exclusion criteria, 381 children with S-ECC and 1220 teeth were included in the study, including 590 primary anterior teeth and 630 primary molars. Data were collected and updated until 31 August 2020. A total of 205 were male (53.81%), and 176 were female (46.19%). The mean age at the time of treatment was 3.49 ± 0.90 years. The average follow-up time was 19.09 ± 11.07 months. During the observation period, 187 children had at least one tooth failed during follow-up with a total of 397 teeth (139 anterior teeth and 258 molars). The average time to failure was 20.38 ± 10.71 months.

**Figure 1:**
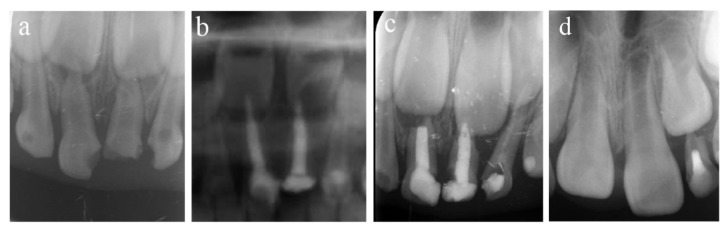
#51 and #61 were treated with pulpectomies under DGA with a 51-month follow-up period (52-month-old male child). (**a**) The radiograph before treatment and #51 revealed periapical radiolucency. (**b**) One week later, the postoperative radiograph showed optimal filling in both teeth. (**c**) Twenty-two months after the treatment, radiography showed physiological root resorption. (**d**) Fifty-one months after treatment, the permanent upper incisors erupted normally. All pulpectomies succeeded.

**Figure 2:**
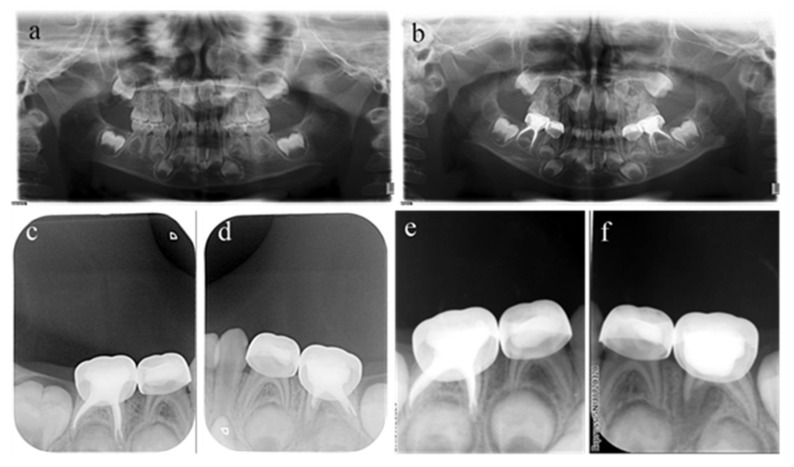
#75 and #85 were treated with pulpectomies under DGA with a 17-month follow-up period (42-month-old female child). (**a**) Preoperative panoramic radiographic showed no periapical radiolucency. (**b**) Two weeks later, the postoperative radiographs showed optimal filling in both teeth; (**c**) Nine months after treatment, the radiograph showed nothing abnormal in #85. (**d**) Nine months after treatment, the filling paste in the root canal of #75 showed partial resorption. (**e**) Seventeen months after treatment, the radiograph showed nothing abnormal in #85. (**f**) Seventeen months after treatment, the filling paste in the root canal of #75 was completely resorbed. Radiolucency was seen around the mesial root as well as pathological root absorption. The pulpectomy in #75 failed but succeeded in #85.

**Figure 3:**
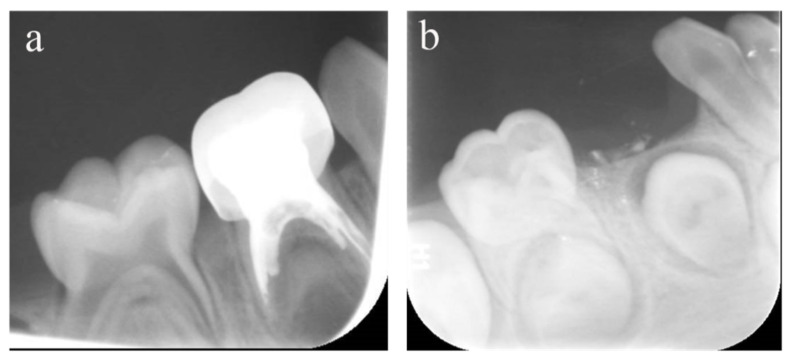
#84 was treated with pulpectomy and filled with ZOE under DGA. The follow-up period was 34 months (31-month-old male child) (**a**) Two weeks after the operation, the radiographic showed that the filling in the distal roots was underfilled. (**b**) Thirty-four months after treatment, #84 was lost early, but a portion of the ZOE particles remained. The pulpectomy in #84 failed.

**Figure 4:**
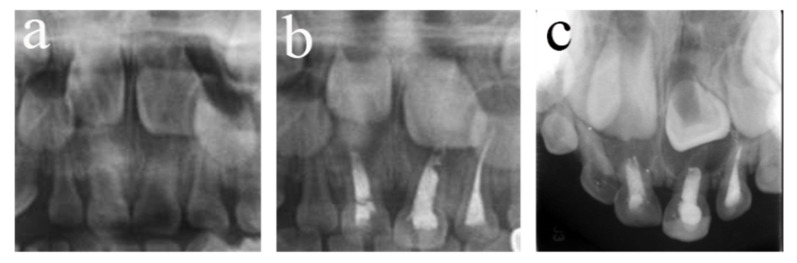
#51, #61, and #62 received pulpectomies under DGA with a 12-month follow-up period (57-month-old female child). (**a**) Preoperative radiograph and #61 revealed periapical radiolucency. (**b**) Two weeks later, the postoperative radiograph showed underfilling in #61 and optimal filling in #51 and #62. (**c**) Radiograph taken 12 months postoperatively showing nothing abnormal about #62 and a periapical radiolucency surrounding the root apical of #51. Moreover, a radicular cyst occurred surrounding the apical root of #61, and the eruption direction of #21 was altered. Pulpectomies in #62 succeeded but failed in #51 and #61.

**Figure 5:**
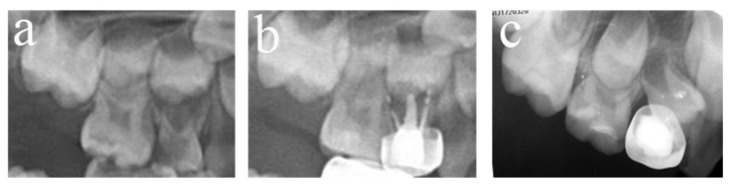
#54 received pulpectomies under DGA with a 34-month follow-up period (65-month-old male child). (**a**) The preoperative radiograph revealed periapical radiolucency surrounding #54. (**b**) Radiograph taken one week postoperatively showing overfilling in the buccal root canals. (**c**) Twenty-six months after the operation, the root was completely absorbed, and the eruption direction of #14 was altered. Pulpectomies in #54 failed.

**Figure 6:**
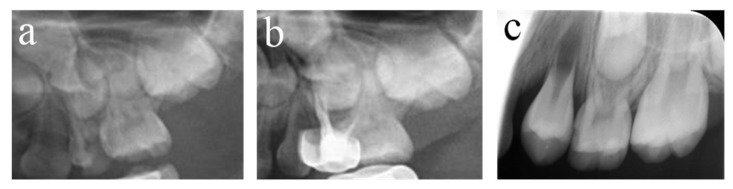
#64 received pulpectomies under DGA with a 32-month follow-up period (63-month-old male child). (**a**) The preoperative radiograph revealed nothing abnormal in the periapical area. (**b**) Radiograph taken one week postoperatively showing optimal filling. (**c**) Thirty-two months after the operation, #64 was lost early, and #24 erupted prematurely. Pulpectomies in #64 failed.

All the failed teeth showed radiological abnormalities (periapical or interradicular radiolucent areas, with or without root resorption). A total of 117 teeth affected the permanent tooth, of which three showed periapical cysts, eight permanently erupted early, and the eruption direction of 10 teeth was altered. Only 125 teeth showed clinical symptoms, among which 75 teeth had abnormal mobility, seven teeth had fistula, 15 teeth had both, three teeth came into residual roots, and 25 teeth were lost early. Figure 1, Figure 2, Figure 3, Figure 4, Figure 5 and Figure 6 showed some photography examples of the pulpectomy teeth with different outcomes.

### 3.1. K-M Survival Analysis

**Figure 7:**
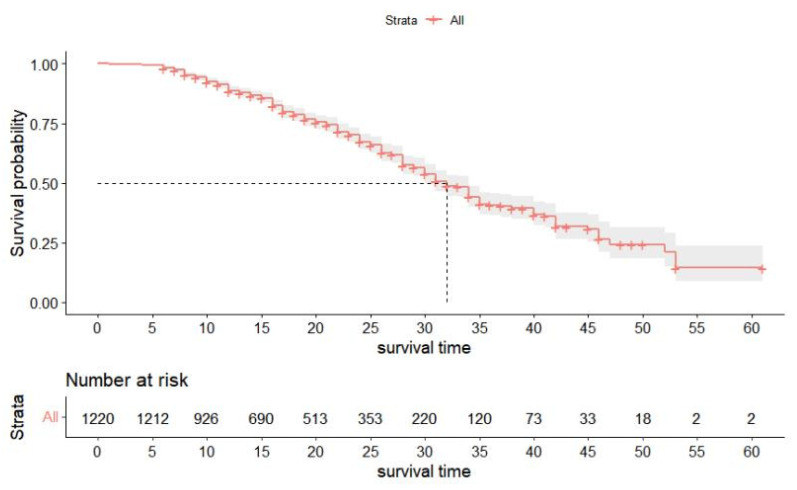
Survival curve of 1220 teeth.

**Figure 8:**
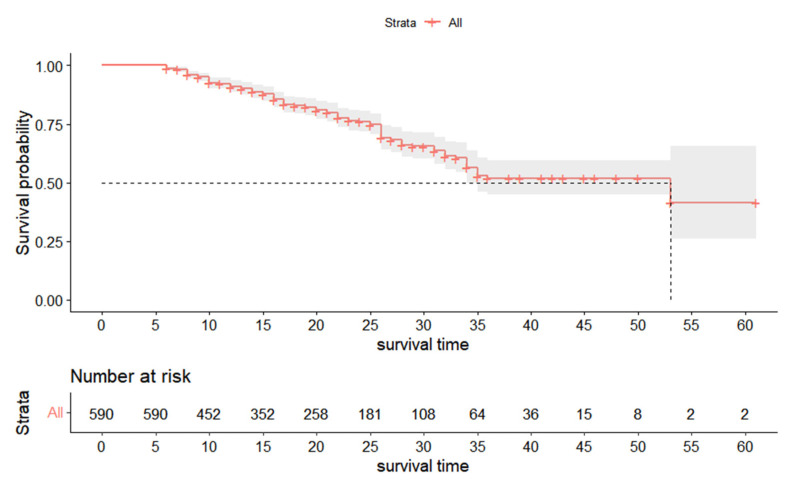
Survival curve of primary anterior teeth (*n* = 590).

**Figure 9:**
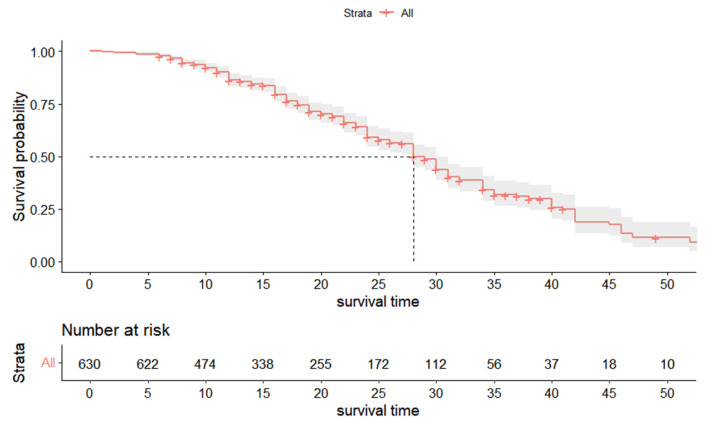
Survival curve of primary molars (*n* = 630).

The results of K-M analysis showed that the median survival time of the 1220 treated teeth was 31 months. The survival curve is shown in Figure 7. The survival rates at 12 months, 18 months, 24 months, 30 months, and 35 months were 88.3%, 78.1%, 66.0%, 52.4%, and 38.5%, respectively. Figure 8 shows the survival curves of 590 primary anterior teeth. The survival rates at 12 months, 18 months, 24 months, 31 months, and 35 months were 90.9%, 82.8%, 76.1%, 63.5%, and 52.9%, respectively, and the median survival time was 35 months. The survival curve of 630 primary molars is shown in Figure 9. The survival rates among primary molars at 12 months, 18 months, 24 months, 31 months, and 35 months were 86.3%, 74.6%, 59.3%, 40.1%, and 31.1%, respectively, and the median survival time was 26 months.

### 3.2. Characteristics of the Teeth Receiving Pulpectomy

The characteristics and proportions of the patients and teeth that underwent failed pulpectomy treatment are shown in Table 1.

### 3.3. Potential Risk Factors for the Primary Tooth by Univariate and Multivariate Analyses

Univariate analysis was performed on the above variables, and the results showed significant differences in age, tooth type, preoperative tooth mobility, soft tissue condition radiological evaluation, and degree of filling (Table 1).

Multivariate analysis was performed for the statistically significant variables in the univariate analysis, and the results are shown in Table 2. The factors affecting the outcomes of pulpectomy of primary teeth are age, tooth types, and preoperative radiological evaluation. The results showed that the older the children were, the greater the risk of treatment failure (HR 1.56, 95% CI 1.09, 2.24). The risk of pulpectomy failure of primary molars was 1.9 times that of primary anterior teeth (95% CI 1.36, 2.65). The failure risk of teeth with abnormal radiological findings before treatment was 1.41 times higher than that of teeth without imaging abnormalities (95% CI 1.74, 3.36).

### 3.4. Potential Risk Factors for the Anterior and Posterior Primary Tooth

Further analysis was performed on anterior teeth and molars separately by multivariate analysis. In this analysis, the independent variables included the age of the children, type of tooth, arch location (molars only), size of the lesions, preoperative tooth mobility, gingiva condition, radiographic evaluation, obturation materials, restoration method (anterior teeth only), and degree of root filling.

### 3.5. Anterior Primary Tooth

The characteristics and proportions of different outcomes among anterior teeth with pulpectomy are shown in Table 3. In the univariate analysis, only the covariates of tooth type and radiographic evaluation showed significant associations (Table 3). In the multivariate model, both tooth type and radiographic evaluation remained statistically associated with pulpectomy failure (Table 4). The chance of pulpectomy failure on primary canine teeth (HR = 0.03, 95% CI 0.01, 0.18) and lateral incisor teeth (HR = 0.51, 95% CI 0.34, 0.80) was lower than that on incisor teeth. The radiological imaging of a tooth with a pathological lesion (HR = 2.75, 95% CI 1.49,5.08) was more likely to have a poor prognosis than a tooth without an abnormal radiographic appearance.

### 3.6. Posterior Primary Tooth

Table 5 shows the characteristics and proportions of the different outcomes among posterior molar teeth that underwent pulpectomy treatment. The covariates included age, arch location, surfaces affected by caries, mobility, gingival condition, radiological evaluation, and the degree of root canal obturation filling and significance in the univariate analysis (Table 5). The risk factors for the molars were age, arch location, tooth surface affected by caries, and radiological evaluation (Table 6) that was significant in the univariate analysis were included in the multivariate analysis. The risk of pulpectomy failure was 1.99 (95% CI 1.24, 3.21) times higher with every one-year increase in patient age. The chance of failure for the mandibular primary molars was 2.42 times higher (95% CI 1.65, 3.56) than that for the maxillary primary molars. Compared with the carious lesions that only affected one surface, the chance of tooth failure with two or more affected surfaces was 3.01 (95% CI 2.09, 7.68) and 3.39 (95% CI 2.00, 9.65) times higher. The radiological imaging of the tooth with a pathological lesion was 2.3 times (95% CI 2.30, 3.89) more likely to have a poor prognosis compared with the tooth without abnormal radiographic appearance.

## 4. Discussion

Pulpectomy in the primary tooth is indicated for teeth with irreversible inflammation or necrosis. This treatment aims to remove debris and infection in the root canals, maintain the tooth’s physiological function, and extend its longevity [16]. Extraction too early may not be a wise option for children. The premature loss of primary teeth may negatively impact masticatory performance and children phonation, which in turn affects the development of general and psychological health [17,18]. The early loss of primary teeth may also cause a space loss between the canines and molars and result in malocclusion and eruption abnormality [17,19,20]. Pulpectomy in the primary tooth can maximize the retention of the nonvital primary tooth.

During the observation period of this study, 1671 children underwent pulpectomy, accounting for 86.54% of the total, and the average number of treated teeth per child was 3.70 ± 2.53. This indicates that pulpectomy in primary teeth accounted for a vast proportion of the pediatric dentist’s normal practice in China. This study showed a good prognosis in the short term, and the survival rate at 12 months reached above 85%, which can effectively control inflammation, relieve clinical symptoms, and restore mastication function. However, the long-term results are not satisfactory, especially in the posterior teeth, which may not survive until natural exfoliation. A recent systematic review of controlled studies found that the clinical and radiographic success rates of pulpectomy in the primary tooth ranged between 64% and 100% among the included studies with a 6–36 months observation period [21]. However, only one study continued over 18 months (36 months) and studied the incisors only. Regarding the short-term prognosis, the result in the present study is consistent with and to some extent higher than other studies. However, for the long-term prognosis, the present study result is lower than the only study with 36 months (52.9% vs. 82%). However, the sample size in that study is much smaller than the present study. Additionally, the survival rate and change tendency is very similar with another study also conducted in China [11].

There are significant differences between the anterior and posterior teeth in root canal anatomy, bite force, and the time of eruption and exfoliation. Therefore, the survival curves of the anterior and posterior teeth were further analyzed separately, and there was a significant difference. Our findings resonate with a previous study suggesting that the median survival period of anterior teeth was significantly longer than that of posterior teeth [11]. The difference is presumed to be induced by the anatomy of the root canal in the primary teeth. The root canal system in molars is much more complex than that in anterior teeth due to intricate microscopic structures such as accessory root canals and lateral root canals. Infection is more likely to reach the root apex and root bifurcation [22], which makes adequate cleaning, shaping, and filling of root canals more difficult.

To explore the potential risk factors that may influence the survival of pulpectomy in primary teeth, this statistical analysis was based on the shared frailty model. The shared frailty model is a random-effects model where the frailties are common (or shared) among groups of individuals and are randomly distributed across groups [23]. In the survival analysis, the Cox regression model is more common, but it assumes that the subjects in the model are independent. However, the survival time of different teeth in the same individual may be correlated. The shared frailty model can effectively reduce the random effects of such correlations [24]. This study showed that the age of the children, tooth type, and radiographic evaluation are risk factors for pulpectomy survival. However, when separating the anterior and posterior teeth, it was found that survival in the anterior tooth was only associated with the tooth type and radiographic conditions. For the posterior tooth, the influencing factors were more complex, including age, tooth location, surfaces affected by caries, and radiological evaluation.

In line with a previous study, it was found that the tooth’s survival rate was significantly negatively correlated with age [11]. The premature primary tooth has a prominent tooth pulp cavity, rich blood supply, and strong repair ability. As the age increases, the volume of the cavity and the repair capacity decrease [25]. In addition, the complexity of the root canal system of primary teeth increases with the physiological or pathological resorption of the root [22].

Regarding the tooth type and position, the prognosis of the primary canine and lateral incisor was significantly better than that of the central incisor. The possible reason is that the maxillary primary central incisor erupted early, and due to the existence of factors such as poor feeding habits, it is one of the predisposing sites of caries. The tooth condition may be relatively serious at the time of treatment. In addition, the eruption and exfoliation of the central incisor occurs much earlier than those of the lateral incisor and canine; thus, tooth resorption occurs earlier, which decreases the effect of treatment and lowers the survival time. The results of multivariate analysis in primary molars showed that the prognosis of maxillary primary molars was more favorable than that of mandibular primary molars, which may be related to the eruption time of the teeth and the anatomy of the root canal. Studies have confirmed that lateral and accessory canals are more prevalent in mandibular molars than in maxillary molars [26]. In addition, there is some overlap between maxillary molars and successive permanent teeth on the radiological image, and small lesions may be relatively more difficult to identify. No statistically significant difference between the first and second primary molars was found in this study, which was consistent with the results of previous studies [27,28].

The number of surfaces affected by caries and the outcome of radiological evaluation are both related to the severity of the infections. After pulpectomy therapy, the resistance of dental hard tissue decreased. The larger the area affected by caries is, the higher the requirements for restoration after treatment and the more factors that need to be considered, such as the sealing ability of the materials, microleakage, and whether a good adjacency relationship can be established for the restoration. That is also why in our daily practice, it is routine to select PMC to restore the primary molars that underwent pulpectomy treatment. Therefore, the method of restoration was not analyzed as a covariate in the multivariate analysis. A previous study also confirmed that the use of PMC could improve the success rate [29]. There was no significant difference in whether the anterior tooth used the strip resin crown. This may indicate that the survival rate does not depend on the restoration method but on the ability to retain the restorations and to avoid defects and microleakage. During the DGA, humidity and technique control could be carried well. In addition, due to the advantages of the strip resin crowns [30], it became a preferred restoration method under DGA treatment when restoring the anterior tooth. Therefore, the number of teeth without strip resin crowns may not be sufficient to detect the difference. One study investigated differences in survival rates between irreversible pulpitis and periapical periodontitis [31], and the findings were consistent with ours. This may be related to the fact that periapical periodontitis of the primary tooth is usually accompanied by root bifurcation lesions, which makes the inflammation more challenging to control [31]. In addition, for teeth with periapical periodontitis, the short time of root canal disinfection under DGA and the lack of medicated disinfection during the interval between visits may be the other reasons for the lower survival rate.

There were also some variables, including mobility, gingival condition, root canal obturation materials, degree of root canal filling, and restoration methods, that did not show a significant correlation with the prognosis of pulpectomy in primary teeth. For the posterior molars, covariates including mobility, gingival condition, and the degree of root canal obturation showed statistical significance in the univariate analysis but not in the multivariate analysis. This indicates that these might have slight influences, but they do not contribute to the core outcome. It can be inferred from the result that radiographic evaluation is more important than clinical symptoms (abnormal ability, gingiva swelling, or fistula) in the primary molars when assessing the prognosis of pulpectomy. Taking radiographic images should be a regular method when necessary and call for the attention of practitioners. The degree of root canal obturation showed a significant difference in the univariate analysis but not in the multivariate analysis. However, in some other studies, the authors considered under- and overfilling to be the reasons for the failure of primary tooth pulpectomy [32,33]. This inconsistency may be due to the difficulty in measuring the primary tooth work length and the deviations of the root canal filling degree of classification.

There are many discussions on root canal obturation materials [34,35,36]. Rabinowitch stated some requirements on the idea of filling materials [37]. Currently, ZOE, calcium hydroxide, and iodoform are the commonly used materials for the filling paste. However, there is still no such primary root filling material that can fulfill all of these requirements, especially in terms of the requirement that the resorption rate should match the physiologic root resorption of the primary teeth [38]. In our clinical, only ZOE paste and Vitapex paste are available. ZOE paste is a manual paste with Zinc oxide powder (0.36 g) and eugenol (0.28 g). ZOE paste had a better inhibitory activity compared with other fillings and a good work time, and it is also easy to mix, cost effective, and insoluble in tissue fluids [39]. The disadvantages of ZOE paste is that the resorption rate of the ZOE particles extruded out of the apical foramen is slow and it alters the path of eruption of succedaneous teeth and causes soft tissue irritation and allergy to eugenol, and exhibits cytotoxicity and neurotoxicity [39,40]. Vitapex is a commercial product which contains 40.4% iodoform, 30.3% calcium hydroxide, and 22.4% silicone. The advantage of Vitapex is its absorbability. The extruded particle from the apex can be absorbed thoroughly and quickly. Meanwhile, the rate of resorption from within the canals is faster than physiological root resorption [39]. A previous meta-analysis compared the success rate between the calcium hydroxide/iodoform paste and ZOE and found no statistically significant difference in the short term (6- and 12-month follow-up) [41]. However, ZOE was shown to have significantly higher success rates at the ≥18-month follow-up [41,42]. In the present study, no significant difference was identified regarding the different filling materials, but a higher failure proportion can still be found in the Vitapex. This may be related to the fact that the absorption rate of Vitapex in the root canal is faster than that of the tooth root, and it is easy to form a cavity in the root canal, which can easily cause reinfection.

This study enriched the survival analysis of primary root canal therapy and the possible potential risk factors. Currently, the research in this field is insufficient, and this study is based on a relatively large sample size and an extended follow-up, so the results of this study are fairly reliable. However, this study still has some limitations that should be emphasized. First, this was a retrospective study. All of the data are extracted from the medical record, and there might be some mis-recording or inaccurate recordings that cannot be identified. Second, the recruited participants came from a convenience sampling and underwent DGA treatment with expert dentists. Thus, the participants and clinicians are not representative of the general population and are prone to selection bias. Third, snacking frequency, feeding and oral hygiene habits or conditions, parental education levels, and socioeconomic background are highly associated with ECC occurrence [43], but in the present study, these factors cannot be controlled because in the previous medical record system, no related information was being recorded. These potential factors may confound the statistical results.

The two-year survival rate of primary tooth pulpectomy is relatively high and stable. The non-vital primary tooth can be maximally retained to rehabilitate the chewing function via pulpectomy. However, during the therapy, its indications should be strictly followed. Attention should be paid to risk factors such as age, tooth position, surfaces affected by caries, and preoperative tooth periapical condition. The AAPD recommends that primary teeth undergoing pulpectomy treatment should take radiographic evaluation biannually [8]. For children with the above risk factors, compliance should be improved to achieve a customized frequency of dental visit, usually once every three to six months.

All of the treatments in the study are completed under DGA. Although it can eliminate the confounder of children’s cooperation, the time for root canal disinfection is shorter, which may be one of the reasons that affect its curative effect. Therefore, how to effectively control the infection of periapical teeth under DGA remains to be further studied. In addition, implementing more high-quality clinical randomized trials in the future can contribute to building a better evidence-based pulpectomy dentistry foundation.

## 5. Conclusions

The survival rate of primary tooth pulpectomy was acceptable but decreased gradually with time. The failure rate of pulpectomy in primary molars is higher than that of primary anterior teeth. When the primary caries has extended to the pulp and resulted in a nonvital lesion, pulpectomy could be an option for the maximum retention of the primary tooth.

## Figures and Tables

**Table 1:** Distribution of different variables based on the outcomes and univariate analysis results.

Factors	S (%)	F (%)	*p* *	HR
Gender				
male	455 (65.4)	241 (34.6)		1
female	368 (70.2)	156 (29.8)	0.77	0.91
Age			**<0.001**	1.85
1–2 years	7 (100)	0 (0.0)		
2–3 years	114 (69.1)	51 (30.9)		
3–4 years	310 (71.8)	122 (28.2)		
4–5 years	288 (62.5)	173 (37.5)		
5–6 years	104 (67.1)	51 (32.9)		
Tooth type				
anterior teeth	451 (76.4)	139 (23.6)		1
molars	372 (59.0)	258 (41.0)	**<0.001**	1.83
Preoperative condition mobility				
physiological	695 (69.6)	303 (30.4)		1
abnormal	128 (57.7)	94 (42.3)	**0.02**	1.52
Soft tissue				
normal	713 (69.0)	320 (31.0)		1
swelling or fistula	110 (58.8)	77 (41.2)	**0.02**	1.55
Radiographic evaluation				
normal	648 (71.2)	262 (28.8)		1
abnormal	175 (56.5)	135 (43.5)	**<0.001**	2.25
Obturation materials				
Vitapex	336 (62.9)	198 (37.1)		1
ZOE	487 (71.0)	199 (29.0)	0.14	0.68
Degree of root filling				
underfilling	202 (70.6)	84 (29.4)		1
optimal fillling	506 (67.3)	246 (32.7)	**0.02**	0.50
overfilling	115 (63.2)	67 (36.8)	0.75	0.90

S–Successful outcomes; F–Failed outcomes; HR–hazard ratio. * Analyzed by shared frailty model, the bold *p* values indicated that the difference was significant (*p* < 0.05).

**Table 2:** Multivariate analysis of primary tooth root canal treatment.

Factor	Coef	SE (Coef)	*p* *	HR	95% CI	
					Lower	Upper
Age	0.45	0.18	**0.02**	1.56	1.09	2.24
Tooth type						
anterior tooth				1		
posterior tooth	0.64	0.17	**<0.001**	1.90	1.36	2.65
Radiological evaluation						
normal				1		
abnormal	0.88	0.17	**<0.001**	2.41	1.74	3.36

Coef—regression coefficient; SE—standard error; HR—hazard ratio; CI—confidence interval. * Analyzed by shared frailty model, the bold *p* values indicated that the difference was significant (*p* < 0.05).

**Table 3:** Anterior tooth distribution of different variables based on the outcomes and univariate analysis results.

Factors	S (%)	F (%)	*p* *	HR
Age			0.53	1.22
1–2 years	7 (100.0)	0 (0.0)		
2–3 years	90 (70.3)	38 (29.7)		
3–4 years	185 (76.8)	56 (23.2)		
4–5 years	135 (75.8)	43 (24.2)		
5–6 years	34 (94.4)	2 (5.6)		
Tooth type				
central incisor	203 (68.8)	95 (31.2)		
lateral incisor	189 (81.6)	45 (18.4)	**<0.001**	0.45
canine	55 (94.8)	3 (5.2)	**<0.001**	0.03
Number of surfaces affected				
Double	187 (79.2)	49 (20.8)	0.32	1
multiple	246 (75.2)	81 (24.8)		1.64
Preoperative tooth mobility				
physiological	368 (80.5)	89 (19.5)	0.09	1
abnormal	83 (62.4)	50 (37.6)		1.63
Soft tissue				
Normal	377 (78.5)	103 (21.5)		1
swelling or Sinus tract	74 (87.3)	36 (12.7)	0.09	1.66
Radiographic evaluation				
Normal	344 (81.1)	80 (18.9)		1
abnormal	107 (64.5)	59 (35.5)	**<0.001**	3.27
Obturation materials				
Vitapex	178 (70.4)	75 (29.6)		1
ZOE	273 (76.4)	64 (23.6)	0.77	0.86
Restoration method				
strip resin crown	357 (75.8)	114 (24.2)		1
resin directly	94 (79.0)	25 (21.0)	0.63	1.22
Degree of root filling				
underfill	113 (81.3)	26 (18.7)		1
optimal fill	292 (73.9)	103 (26.1)	0.63	1.40
Overfill	46 (82.1)	10 (18.9)	0.67	1.27

S—Successful outcomes; F—Failed outcomes; HR—Hazard ratio. * Analyzed by shared frailty model, the bold *p* values indicated that the difference was significant (*p* < 0.05).

**Table 4:** Multivariate analysis of the anterior teeth.

	Coef	SE (Coef)	*p* *	HR	95% CI	
					Lower	Upper
Type of primary tooth						
Central incisor				1		
Lateral incisor	−0.65	0.22	**<0.001**	0.51	0.34	0.80
Canine	−3.41	0.86	**<0.001**	0.03	0.01	0.18
Radiological evaluation						
Abnormal				1		
Normal	1.01	0.31	**<0.001**	2.75	1.49	5.08

Coef—regression coefficient; SE—Standard error; HR—hazard ratio; CI—confidence interval. * Analyzed by shared frailty model, the bold *p* values indicated that the difference was significant (*p* < 0.05).

**Table 5:** Posterior tooth distribution of different variables based on the outcomes and univariate analysis results.

Factors	S (%)	F (%)	*p* *	HR
Age			**0.001**	2.16
2–3 years	24 (64.9)	13 (35.1)		
3–4 years	125 (65.4)	66 (34.6)		
4–5 years	153 (54.1)	130 (45.9)		
5–6 years	70 (58.8)	49 (41.2)		
Tooth location				
maxillary molar	142 (67.6)	68 (32.4)		1
mandibular molar	230 (54.8)	190 (45.2)	**<0.001**	2.00
Tooth type				
1st molar	165 (52.9)	147 (47.1)		1
2nd molar	207 (65.1)	111 (34.9)	**0.04**	0.72
Number of surfaces affected				
Single	110 (66.3)	56 (33.7)		1
Double	157 (56.5)	121 (43.5)	**0.002**	2.66
Multiple	100 (56.5)	77 (43.5)	**<0.001**	4.29
Preoperative tooth mobility				
physiological	327 (60.4)	214 (39.6)		1
Abnormal	45 (50.6)	44 (49.4)	**0.03**	1.94
Soft tissue				
Normal	336 (60.8)	217 (39.2)		1
swelling or fistula	36 (46.8)	41 (53.2)	**0.05**	1.83
Radiographic evaluation				
Normal	304 (62.6)	182 (37.4)		1
Abnormal	68 (47.2)	76 (52.8)	**<0.001**	2.39
Obturation materials				
Vitapex	158 (56.2)	123 (43.8)		1
ZOE	214 (61.3)	135 (38.7)	0.14	0.58
Degree of root filling				
Underfilling	89 (60.5)	58 (39.5)		1
optimal filling	214 (59.9)	143 (40.1)	**0.01**	0.38
Overfilling	69 (54.8)	57 (45.2)	0.15	0.57

S—Successful outcomes; F—Failed outcomes; HR—Hazard ratio. * Analyzed by shared frailty model, the bold *p* values indicated that the difference was significant (*p* < 0.05).

**Table 6:** Multivariate analysis of the posterior tooth.

Factor	Coef	SE (Coef)	*p* *	HR	95%CI	
					Lower	Upper
Age	0.69	0.24	**0.005**	1.99	1.24	3.21
Location:						
Maxillary				1		
Mandibular	0.88	0.20	**<0.001**	2.42	1.65	3.56
Number of surfaces affected						
One				1		
Two	1.39	0.33	**<0.001**	4.01	2.09	7.68
Multiple	1.48	0.40	**<0.001**	4.39	2.00	9.65
Radiological evaluation						
Normal				1		
Abnormal	0.83	0.27	**0.002**	2.30	1.36	3.89

Coef—regression coefficient; SE—standard error; HR—hazard ratio; CI—confidence interval. * Analyzed by shared frailty model, the bold *p* values indicated that the difference was significant (*p* < 0.05).

## Data Availability

The data presented in this study are available on request from the corresponding author. The data are not publicly available due to the policy by College of Stomatology, Xi’an Jiaotong University.
